# Charge–Discharge Mechanism of High‐Entropy Co‐Free Spinel Oxide Toward Li^+^ Storage Examined Using Operando Quick‐Scanning X‐Ray Absorption Spectroscopy

**DOI:** 10.1002/advs.202201219

**Published:** 2022-05-26

**Authors:** Xu‐Feng Luo, Jagabandhu Patra, Wei‐Tsung Chuang, Thi Xuyen Nguyen, Jyh‐Ming Ting, Ju Li, Chih‐Wen Pao, Jeng‐Kuei Chang

**Affiliations:** ^1^ National Synchrotron Radiation Research Center, Hsin‐Ann Road Hsinchu Science Park Hsinchu 30076 Taiwan; ^2^ Department of Materials Science and Engineering National Yang Ming Chiao Tung University 1001 University Road Hsinchu 30010 Taiwan; ^3^ Hierarchical Green‐Energy Materials (Hi‐GEM) Research Center National Cheng Kung University 1 University Road Tainan 70101 Taiwan; ^4^ Department of Materials Science and Engineering National Cheng Kung University 1 University Road Tainan 70101 Taiwan; ^5^ Department of Nuclear Science and Engineering and Department of Materials Science and Engineering Massachusetts Institute of Technology 77 Massachusetts Avenue Cambridge MA 02139 USA

**Keywords:** charge storage mechanism, high energy density, high‐entropy anode, Li‐ion batteries, lithiation/delithiation

## Abstract

Transition metal high‐entropy oxides (HEOs) are an attractive class of anode materials for high‐performance lithium‐ion batteries (LIBs). However, owing to the multiple electroactive centers of HEOs, the Li^+^ storage mechanism is complex and debated in the literature. In this work, operando quick‐scanning X‐ray absorption spectroscopy (XAS) is used to study the lithiation/delithiation mechanism of the Cobalt‐free spinel (CrMnFeNiCu)_3_O_4_ HEO. A monochromator oscillation frequency of 2 Hz is used and 240 spectra are integrated to achieve a 2 min time resolution. High‐photon‐flux synchrotron radiation is employed to increase the XAS sensitivity. The results indicate that the Cu^2+^ and Ni^2+^ cations are reduced to their metallic states during lithiation but their oxidation reactions are less favorable compared to the other elements upon delithiation. The Mn^2+/3+^ and Fe^2+/3+^ cations undergo two‐step conversion reactions to form metallic phases, with MnO and FeO as the intermediate species, respectively. During delithiation, the oxidation of Mn occurs prior to that of Fe. The Cr^3+^ cations are reduced to CrO and then Cr^0^ during lithiation. A relatively large overpotential is required to activate the Cr reoxidation reaction. The Cr^3+^ cations are found after delithiation. These results can guide the material design of HEOs for improving LIB performance.

## Graphical abstract

The charge‐storage mechanism of Cobalt‐Free high‐entropy oxide is studied using a novel operando quick‐scanning X‐ray absorption spectroscopy (XAS) technique. High‐photon‐flux synchrotron radiation is employed to increase XAS sensitivity.XAS data are acquired in on‐the‐fly mode using a monochromator oscillation frequency of 2 Hz. Valence/coordination state variations and multiple transition steps of the constituent species upon charging/discharging are investigated in detail.

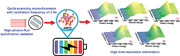

## 1 Introduction

The rising demand for portable electronic devices, electric vehicles, and large‐scale energy storage has spurred innovation in the field of lithium‐ion batteries (LIBs). Many approaches have been proposed for improving LIB performance.^[^ ^1^, ^2^, ^3^, ^4^ ^]^ Increases in the specific capacity and rate capability of anode materials are particularly desirable for next‐generation LIBs because the conventional graphite electrode has a limited capacity and unsatisfactory high‐rate performance.^[^ ^5^ ^]^ Lithium (Li) metal is an appealing candidate anode material due to its low potential and extremely high capacity (theoretically 3860 mAh g^−1^).^[^ ^6^, ^7^ ^]^ However, the dendrite formation and high reactivity of Li metal raise concerns about safety and short cycle life, which must be addressed for practical applications.^[^ ^8^, ^9^ ^]^ Conversion‐type anodes are another attractive option for high‐energy‐density LIBs.^[^ ^10^ ^]^ The conversion reactions of transition metal compounds (e.g., oxides, sulfides, nitrides) with Li^+^ ions form Li*_n_* X (X = O, S, N, etc.) and metallic particles.^[^ ^11^ ^]^ Multielectron transfer reactions give the anodes large specific capacities.^[^ ^12^ ^]^ In addition, the relatively high redox potential of the conversion reactions avoids the formation of metallic Li during battery charging. However, conventional conversion‐type anodes suffer from low cycling stability, which is associated with severe crystallinity and volume changes during lithiation/delithiation.^[^ ^13^ ^]^ In addition, their low electronic/ionic conductivities result in inferior rate capability.^[^ ^10^, ^13^ ^]^ Transition metal high‐entropy oxides (HEOs) are a new class of conversion‐type anode that integrates five (or more) elements into a single phase to increase the configurational entropy (i.e., *S*_config_ ≥ 1.5 *R*).^[^ ^14^, ^15^ ^]^ The entropy‐derived phase stabilization effects can improve electrode reversibility and cycleability.^[^ ^16^, ^17^, ^18^ ^]^ Moreover, the HEO lattice is distorted due to the different sizes of the constituent atoms, which creates a lattice residual stress that can alter material properties.^[^ ^19^ ^]^ Rock‐salt‐structure HEO anodes have been proposed since 2018.^[^ ^16^, ^20^, ^21^ ^]^ The Co_0.2_Cu_0.2_Mg_0.2_Ni_0.2_Zn_0.2_O HEO exhibited a reversible capacity of 600 mAh g^−1^ and great cycling stability of over 500 cycles.^[^ ^16^ ^]^ Recently, spinel‐structure HEOs have attracted much attention because of their two Wyckoff sites and abundant oxygen vacancies, which are favorable for reversible Li^+^ storage.^[^ ^17^, ^22^, ^23^ ^]^ A spinel (CrMnFeCoNi)_3_O_4_ anode synthesized via a surfactant‐assisted hydrothermal method was found to have a great charge–discharge capacity of 1235 mAh g^−1^ and 90% capacity retention after 200 cycles.^[^ ^22^ ^]^ It is worth noting that most of the reported HEO anodes include cobalt (Co), which provides high conductivity and ensures stable charge–discharge performance.^[^ ^16^, ^20^, ^22^, ^24^ ^]^ However, Co is scarce, toxic, and expensive, and is vulnerable to geopolitical interference and global supply problems.^[^ ^25^ ^]^ The development of Cobalt (Co)‐free HEOs (where Co is replaced by earth‐abundant elements) is thus of great importance.^[^ ^26^ ^]^

The Li^+^ uptake/release mechanism of HEOs is complex due to their multiple electroactive centers and thus debated in the literature. Breitung et al. proposed that the redox reactions of Co^2+^ and Cu^2+^ in the rock salt (Co_0.2_Cu_0.2_Mg_0.2_Ni_0.2_Zn_0.2_)O were mainly responsible for the reversible capacity, whereas the other elements helped to stabilize the crystal structure.^[^ ^16^ ^]^ However, for the same HEO, Cui et al. found that the MgO that formed after the first lithiation process was the only inactive component, which prevented the other active species from aggregation and degradation.^[^ ^20^ ^]^ Different from these two papers, Quartarone et al. indicated that the metallic Zn and Mg produced from this HEO after conversion reactions further underwent alloying reactions with Li^+^.^[^ ^21^ ^]^ Chang et al. confirmed, via a combined scanning transmission electron microscopy/electron energy‐loss spectroscopy technique, that all the cations in the spinel (CrMnFeCoNi)_3_O_4_ HEO actively participated in the lithiation/delithiation redox reactions. In addition, a phase segregation of the pristine HEO into new Cr*_x_* Fe_3−_ *_x_* O_4_ and LiNi*_x_* Co_1−_ *_x_* O_2_ spinel phases was observed.^[^ ^24^ ^]^ Accordingly, the charge storage mechanism of HEOs seems to vary with crystal structure and chemical composition. The detailed HEO redox mechanism, which is not fully understood, should be further examined to facilitate electrode material design for improving battery performance. The analytical tools are crucial for examining the reaction mechanisms. It is difficult to detect short‐range ordered nanocrystals using X‐ray diffraction (XRD) due to the limitation of the X‐ray coherence length.^[^ ^27^, ^28^ ^]^ Electron diffraction has a poor resolution for determining the precise *d*‐spacing. Thus, it is difficult to distinguish different kinds of oxide in an HEO sample based on electron diffraction patterns.^[^ ^29^ ^]^ X‐ray photoelectron spectroscopy (XPS) probes only the outermost surface chemistry, not the bulk properties, of electrodes. Therefore, limited information can be obtained. Moreover, ex situ analyses usually face contamination problems; air and moisture exposure can affect experimental results, especially for highly lithiated electrodes with strong reactivity.^[^ ^30^, ^31^, ^32^ ^]^ These problems may explain the inconsistent redox reaction mechanisms reported in the literature. Operando analysis is thus required to probe the electrode material variation during charging/discharging in real time. Operando X‐ray absorption spectroscopy (XAS) is sensitive to the local environments and electronic structures of absorbing atoms, making it suitable for mechanistic investigations. The high penetrability of hard X‐ray allows XAS to examine all constituent elements within an HEO electrode.

**Scheme 1:**
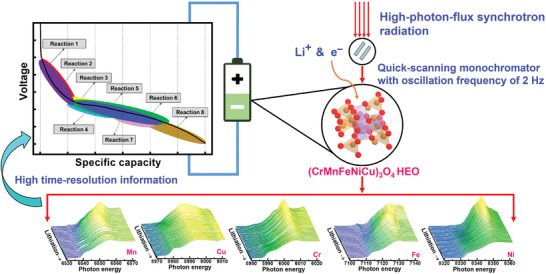
Using operando quick‐scanning XAS to examine valence/coordination state variations, transition steps, redox sequence, reversibility, and redox overpotential of multiple electroactive centers in (CrMnFeNiCu)_3_O_4_ HEO electrode.

In the present work, the charge storage mechanism of a cobalt‐free spinel (CrMnFeNiCu)_3_O_4_ HEO is studied using XAS. Conventional XAS operated in monochromator step‐by‐step scan mode usually requires dozens of minutes to acquire a single spectrum. Thus, it is difficult to determine the detailed material changes and short‐lived intermediate species during electrochemical lithiation/delithiation. Therefore, a quick‐scanning monochromator operated in on‐the‐fly mode^[^ ^33^ ^]^ is employed in this study. This synchrotron facility is operated at an electron energy of 3 GeV with an electron beam current of 400 mA, providing a photon flux of 3 × 10^11^ photons per second at 10 keV. Thus, it can be used to conduct a high‐sensitivity and high‐time‐resolution operando XAS analysis. The quick‐scanning XAS is first used to study HEO electrodes during charging/discharging. The XAS spectra are acquired in transmission mode using a monochromator oscillation frequency of 2 Hz (i.e., two complete spectra are acquired per second). A total of 240 spectra are integrated to ensure a good signal‐to‐noise ratio and achieve a 2 min time resolution. The valence/coordination state variations, multiple transition steps, redox sequence, reversibility, and redox overpotential of Cr, Mn, Fe, Ni, and Cu species in the HEO electrode are examined, as illustrated in **Scheme** **1**.

## 2 Results and Discussion

### 2.1 Material and Electrochemical Properties

**Figure 1:**
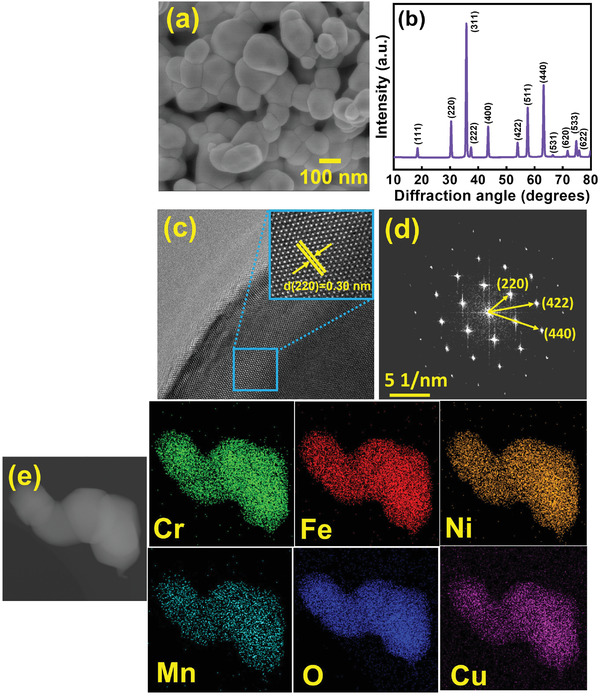
a) SEM image, b) XRD pattern, c) high‐resolution TEM image, d) SAED pattern, and e) HAADF image with EDS elemental mapping data of (CrMnFeNiCu)_3_O_4_ HEO powder.

**Figure** **1**a shows the scanning electron microscopy (SEM) morphology of the obtained (CrMnFeNiCu)_3_O_4_ powder, which has a spherical shape with a diameter ranging from 100 to 200 nm. The XRD pattern of the sample is shown in Figure 1b. The diffraction peaks at ≈18°, 30°, 36°, 37°, 43°, 54°, 57°, 63°,66°, 71°, 74°, and 76° correspond to the (111), (220), (311), (222), (400), (422), (511), (440), (531), (620), (533), and (622) planes, respectively, of a spinel structure that belongs to a *Fd‐3m* space group (JCPDS no. 22‐1084).^[^ ^22^ ^]^ No impurity peak was found, confirming the formation of a single‐phase HEO. Transmission electron microscopy (TEM) analyses were also conducted. A lattice fringe with a spacing of 0.30 nm, which corresponds to the (220) plane distance, can be seen in the high‐resolution TEM image (Figure 1c). The *d*‐spacing values calculated from the selected area electron diffraction (SAED) pattern in Figure 1d are consistent with the XRD data. A high‐angle annular dark‐field (HAADF) image and energy‐dispersive X‐ray spectroscopy (EDS) elemental mapping data are given in Figure 1e) A homogeneous distribution of all elements was confirmed.

**Figure 2:**
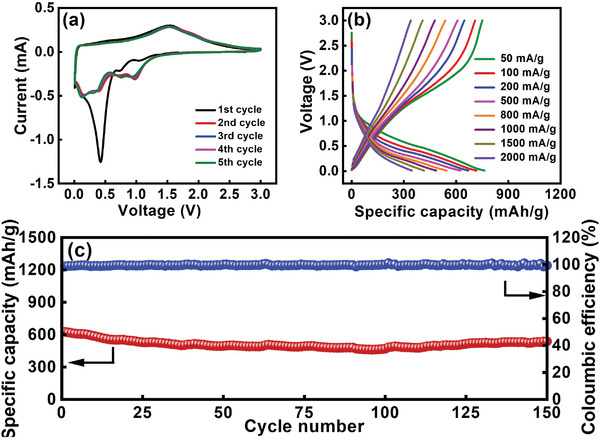
a) CV curves recorded at potential scan rate of 0.1 mV s^−1^, b) charge–discharge curves measured at various current rates, and c) cycling stability data of HEO electrode measured at 500 mA g^−1^.

A cyclic voltammetry (CV) scan was carried out to examine the electrochemical behavior of the (CrMnFeNiCu)_3_O_4_ electrode. As shown in **Figure** **2**a, in the first negative (i.e., lithiation) scan, cathodic peaks appear at ≈1.06, 0.74, 0.43, and 0.16 V. These peaks are attributed to electrolyte decomposition (and thus solid electrolyte interphase (SEI) formation), stepwise conversion reactions of the HEO, and Li_2_O formation.^[^ ^21^, ^34^ ^]^ In the subsequent positive scan, a broad anodic peak centered at ≈1.5 V was observed, reflecting a reconversion (i.e., Li^+^ release) reaction of the electrode.^[^ ^35^, ^36^ ^]^ It is noted that the cathodic charge is larger than the anodic charge in the first cycle, which is ascribed to the SEI formation and irreversible trapping of Li^+^ within the electrode. From the second cycle onward, the CV curves almost overlap, indicating great electrochemical lithiation/delithiation reversibility of the HEO electrode.

Galvanostatic charge–discharge tests were performed to evaluate the Li^+^ storage properties of the HEO electrode. Figure S1 (Supporting Information) shows the initial three charge–discharge cycles recorded at 50 mA g^−1^. Consistent with the CV data, a clear plateau below 0.5 V was observed in the first lithiation (defined as charging in this work) curve. The first‐cycle Coulombic efficiency (CE) was ≈63%. The charge–discharge properties became steady after the first cycle, with a CE of ≈99%. Figure 2b shows the charge–discharge profiles of the HEO electrode recorded at various current rates. At 50 mA g^−1^, the measured specific capacity was 750 mAh g^−1^. With increasing applied current, the specific capacity gradually decreased due to kinetic limitations.^[^ ^11^, ^37^ ^]^ However, at 2000 mA g^−1^, a specific capacity of 340 mAh g^−1^ was still obtained, corresponding to a decent capacity retention of 45% compared to the value measured at 50 mA g^−1^. We expect that an HEO||LiNi_0.8_Co_0.1_Mn_0.1_O_2_ full cell could have a specific energy (based on the weight of electroactive materials) about 10% higher than that of a counterpart cell with a conventional graphite negative electrode (with a capacity of ≈300 mAh g^−1^ and an average potential of ≈0.2 V vs Li^+^/Li). The tap densities of the HEO and graphite powder are ≈1.4 and ≈1.0 g cm^−3^, respectively. Therefore, in terms of volumetric performance, the merit of the HEO negative electrode is more pronounced. Figure 2c shows the great cycling stability of the HEO electrode, which shows almost no capacity decay after 150 cycles. The results suggest that (CrMnFeNiCu)_3_O_4_ HEO is a promising anode material for next‐generation LIBs.

Figure S2 (Supporting Information) compares the charge–discharge curves of the HEO electrodes with Cu foil and graphite paper current collectors since we use the former for charge–discharge performance evaluation and the latter for XAS study. The results indicate that the two electrodes showed similar electrochemical behavior at 150 mA g^−1^.

### 2.2 Valence and Coordination States of Transition Metal Elements within (CrMnFeNiCu)_3_O_4_

**Figure 3:**
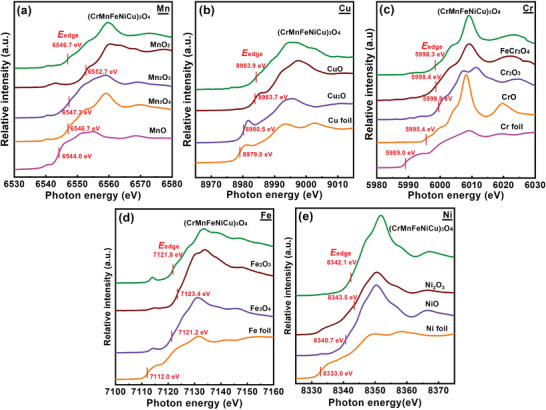
Normalized a) Mn, b) Cu, c) Cr, d) Fe, and e) Ni K‐edge XAS spectra of as‐synthesized HEO powder and reference compounds.

XAS was conducted to examine the valence state of each constituent element in the as‐synthesized HEO. In general, the energy position of the absorption edge correlates with the types of atoms and the valence states. Moreover, the shapes of the XAS profiles are indicative of the coordination structures.^[^ ^38^, ^39^ ^]^ The obtained XAS data of the HEO, together with the spectra of various reference materials, are shown in **Figure** **3**. The XRD pattern in Figure 1b confirms that the synthesized HEO has a single‐phase spinel structure. However, the oxidation states of the individual cations need further identification. Figure 3a shows the Mn K‐edge spectrum of the (CrMnFeNiCu)_3_O_4_ sample. The energy positions of the absorption edge (*E*_edge_), marked in the figure, were determined from the peaks in the first‐derivative plot.^[^ ^40^, ^41^ ^]^ As shown, all the Mn samples show weak pre‐edge peaks at ≈6540 eV. The profile of the HEO sample is composed of a white line at ≈6559.7 eV and a shoulder at ≈6553.7 eV. These features are similar to those of the Mn_3_O_4_ reference. In addition, the HEO sample exhibits an *E*_edge_ value of 6546.7 eV, which is identical to that of Mn_3_O_4_. A mixed Mn^2+/3+^ valence in the HEO was thus identified. The Cu K‐edge spectra of the HEO sample and Cu foil, Cu_2_O, and CuO references are shown in Figure 3b. The *E*_edge_ value of the HEO is 8983.9 eV, which is close to that (8983.7 eV) of CuO. Accordingly, Cu^2+^ seems to be the dominant species within (CrMnFeNiCu)_3_O_4_. Figure 3c shows the Cr K‐edge XAS spectra. With increasing oxidation state, *E*_edge_ shifts toward higher energy, from 5989.0 eV for metallic Cr, 5995.4 eV for CrO, to 5998.4 eV for FeCr_2_O_4_ and 5998.8 eV for Cr_2_O_3_. The HEO sample shows an *E*_edge_ value of 5998.3 eV, which is indicative of the Cr^3+^ state. Moreover, the XAS profile of the HEO consists of a white line at 6009.1 eV, a shoulder peak at 6004.0 eV, and a pre‐edge peak, which well coincide with the spectrum of spinel FeCr_2_O_4_. The results confirm that the Cr element mainly exists as Cr^3+^ in the HEO. The Fe K‐edge spectra of the HEO sample and the Fe, Fe_3_O_4_, and Fe_2_O_3_ reference compounds are shown in Figure 3d. While the *E*_edge_ of Fe foil locates at 7112.0 eV, the absorption edge shifts toward higher energy with increasing the valence state. The Fe_3_O_4_ and Fe_2_O_3_ have *E*_edge_ values of 7121.2 and 7123.4 eV, respectively. The *E*_edge_ of the HEO is 7121.8 eV, which is close to that of spinel Fe_3_O_4_. In addition, the HEO and Fe_3_O_4_ profile shapes are similar, with a shoulder peak at ≈7127 eV. Therefore, the Fe cation in the HEO is identified as a mixed Fe^2+/3+^ state. In the Ni K‐edge spectra, as shown in Figure 3e, the *E*_edge_ values of metallic Ni, NiO, and Ni_2_O_3_ are 8333.0, 8340.7, and 8343.5 eV, respectively. The HEO sample has an *E*_edge_ at 8342.1 eV. It is noted that the HEO shows a shoulder peak at ≈8347 eV, similar to that of NiO. The data thus suggest that the Ni cation within (CrMnFeNiCu)_3_O_4_ is mainly Ni^2+^.

**Figure 4:**
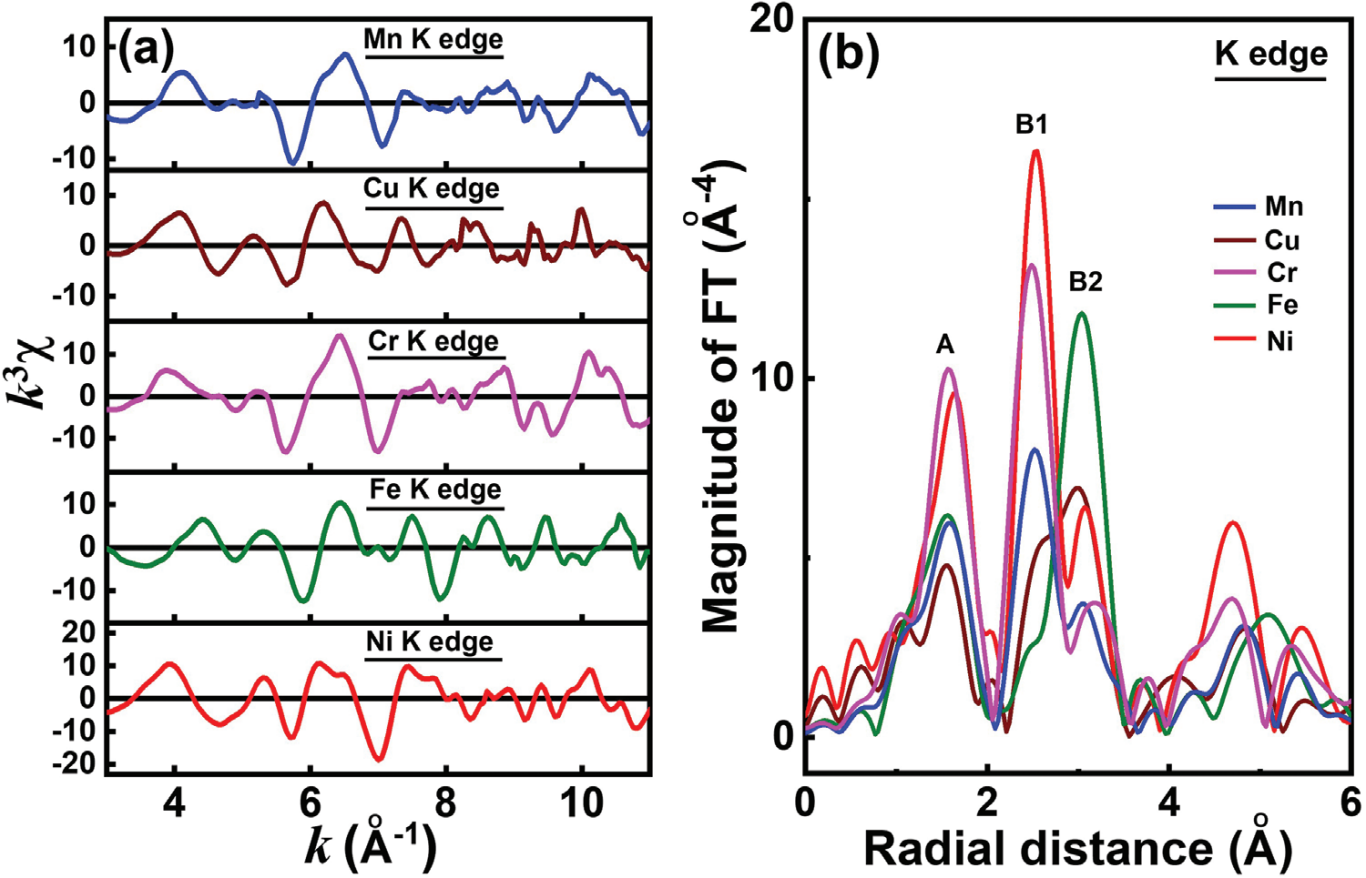
a) K‐edge *k* ^3^‐weighted EXAFS spectra and b) Fourier transform magnitude spectra of all constituent elements in HEO.

The extended X‐ray absorption fine structure (EXAFS) analysis was used to examine the local coordination environments of the HEO constituent elements. **Figure** **4**a,b shows the K‐edge *k* ^3^‐weighted EXAFS and Fourier transform (FT) magnitude spectra, respectively. The selected *k* range for FT is 3–11.0 Å^−1^. All the FT spectra exhibit two major signals (A and B), which correspond to the metal—oxygen bond and metal—metal bond, respectively. There are two subpeaks (B1 and B2) of the signal B, which are associated with the scattering by the metal atoms at octahedral and tetrahedral sites, respectively. This is a typical EXAFS feature for a spinel‐type compound.^[^ ^42^ ^]^ Because the scattering amplitude and phase of the electron waves from the HEO metal atoms are similar, it is difficult to identify the exact coordination structures and scattering atoms from data fitting of the signal B. The similar features of the FT spectra (Figure 4b) suggest that the constituent elements in the HEO are randomly distributed in the crystal lattice. However, the higher signal A intensity of the Ni and Cr spectra indicates that these two elements prefer to occupy the octahedral site (rather than the tetrahedral site) to some extent. It is noted that transmission mode, which can reveal the bulk properties of a material, was used in our XAS study. Compared to the XPS surface analyses (usually at a depth of a few nanometers) usually reported in the literature, we believe that the XAS technique can more precisely depict the oxidation states and site occupancies of individual elements.

### 2.3 Ex Situ XRD and XAS Investigation

The HEO anode undergoes complex conversion reactions during charge–discharge, as shown by the CV curves in Figure 2a. The coexistence of five kinds of cation complicates the charge storage mechanism. The reduction and oxidation reaction products are nanoscale, defective, and poorly crystalline, and thus difficult to probe via XRD.^[^ ^16^, ^18^, ^20^, ^43^ ^]^ As shown in Figure S3 (Supporting Information), the diffraction peaks of the pristine spinel HEO vanish after the first lithiation process and never recover upon delithiation (defined as discharging in this work). This means that the long‐range lattice ordering was broken. Because the short‐range ordered crystals are barely detectable with XRD, the data provide little information on the charge–discharge mechanism. To monitor the valence state variations of the constituent elements and understand the electrochemical reaction routes, XAS analyses are necessary. Figure S4 (Supporting Information) shows the ex situ XAS spectra of the HEO electrodes at lithiation and delithiation states. The spectra of the pristine electrode are also presented for comparison. In the Mn and Fe spectra, a clear negative shift upon lithiation and then a reverse shift upon delithiation are found. A relatively small peak shift is observed for the ex situ Cr spectra. In contrast, the Cu and Ni cations are reduced during lithiation, but the reversibility seems to be poor. Of note, such limited valence state variation of Mn, Fe, and Cr cannot contribute to the high reversible capacity of ≈750 mAh g^−1^ (see Figure 2b) given the chemical composition of the HEO (in Table S1, Supporting Information). The ex situ XAS data thus cannot be used to accurately assess the charge–discharge reactions of the (CrMnFeNiCu)_3_O_4_ electrode. A more reliable analytical technique is required.

### 2.4 Operando XAS Study

**Figure 5:**
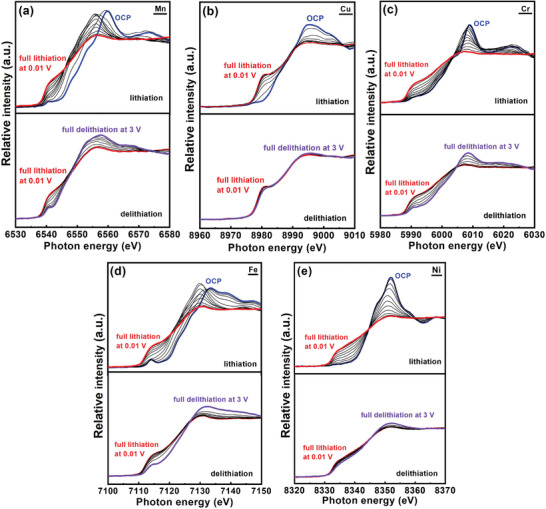
Operando a) Mn, b) Cu, c) Cr, d) Fe, and e) Ni XAS spectra of HEO electrode measured during first charging and discharge at 150 mA g^−1^. The interval between spectra is 100 mAh g^−1^.

Operando XAS measurements of the HEO electrode during galvanostatic charge–discharge at 150 mA g^−1^ were performed. **Figure** **5** shows the variations of the K‐edge spectra for all the constituent elements in the HEO. Upon lithiation, the absorption edges of all spectra gradually shift toward lower energy, indicating the occurrence of reduction reactions during Li^+^ uptake. Comparison of the XAS spectra of the fully lithiated HEO electrodes measured using the ex situ and operando methods is shown in Figure S4 (Supporting Information). All the operando spectra are close to the profiles of the metal foil standards, confirming the formation of metallic Mn, Cu, Cr, Fe, and Ni after lithiation at 0.01 V. However, the ex situ spectra, especially for Mn, Cr, and Fe, show the absorption edges as more positive than those of the operando spectra. Lithiated compounds are highly reactive to air and moisture.^[^ ^30^, ^31^, ^32^ ^]^ Moreover, once the applied potential is unloaded, electrode self‐discharge reactions may occur. A certain degree of undesirable oxidation occurred for the ex situ sample, leading to the positive shift in the spectra. As also shown in Figure 5, during delithiation, the Mn, Cr, and Fe spectra clearly move toward the positive side with increasing discharge capacity, indicating progressive oxidation reactions. Marginal oxidation was observed for Ni. However, the absorption edge of Cu was fixed throughout the delithiation, indicating irreversibility. The ex situ and operando spectra of the delithiated HEO electrodes are compared in Figure S5 (Supporting Information). The energy difference between the two samples is less than that of the lithiated samples shown in Figure S4 (Supporting Information), probably due to the lower moisture/oxygen sensitivity of the delithiated electrodes. According to Figures S4 and S5 (Supporting Information), the ex situ data may be misleading. Using an operando approach is crucial for accurately investigating the reaction mechanism of the HEO electrode during Li^+^ uptake/release.

To assess the valence variations of the elements in (CrMnFeNiCu)_3_O_4_ during charging/discharging, the energy position at 20% of the height of the edge jump was determined as an index of the oxidation state. The energy shift (∆*E*) of the operando XAS spectra was calculated as

(1) $$\Delta E=E\left(0.2\right)-E_{0}\left(0.2\right)$$

**Figure 6:**
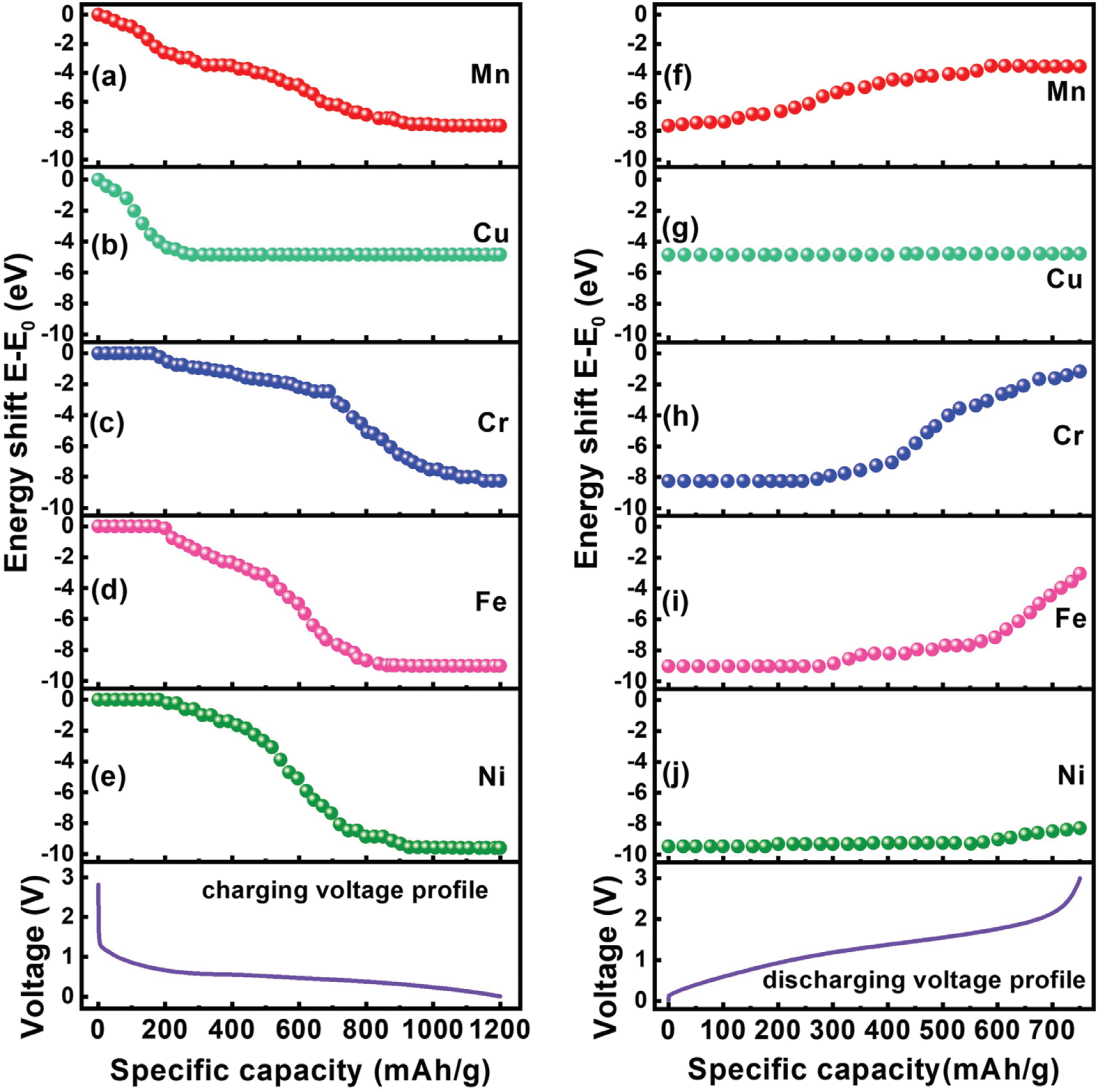
Capacity‐dependent energy shifts of various XAS spectra and voltage profile of HEO electrode during first a–e) charging and f–j) discharging.

**Figure 7:**
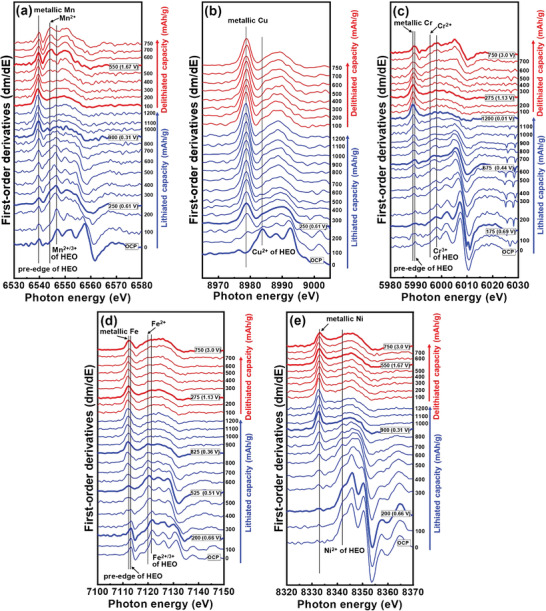
First‐derivative curves of operando a) Mn, b) Cu, c) Cr, d) Fe, and e) Ni XAS spectra of HEO electrode measured during charging and discharge at 150 mA g^−1^.

*E* (0.2) and *E*_0_ (0.2) are the energy positions at 20% of the height of the edge jump of the XAS spectra measured at a particular state of charge and before charging, respectively. As shown in **Figure** **6**a, Mn^2+/3+^ is immediately reduced after charging. There are two steps in the energy shift of Mn, one until 250 mAh g^−1^ (from ≈2.0 to ≈0.6 V) and the other from 250 to 900 mAh g^−1^ (≈0.61 to ≈0.31 V). The first‐derivative curves of the K‐edge spectra were used to examine the coordination structure change of the HEO electrode. As exhibited in the Mn spectra in **Figure** **7**a, a shoulder peak appears at 6543.4 eV upon charging, which is associated with the formation of MnO. After 250 mAh g^−1^, the metallic Mn signal at ≈6539.2 eV begins to intensify, and the oxide features gradually decrease. This suggests the conversion reactions of mixed Mn^2+/3+^ and Mn^2+^ to Mn^0^. Figure 6b shows that Cu^2+^ also starts to be reduced right after charging and contributes to the initial ≈250 mA g^−1^ capacity before the potential reaches ≈0.61 V. According to Figure 7b, Cu^2+^ undergoes a single‐step conversion to form Cu^0^. The Cu^2+^ signal at 8983.9 eV diminishes, and the peak of metallic Cu at 8979 eV increases until a charge capacity of ≈250 mA g^−1^. Afterward, there are no obvious changes in the spectral features. For Cr spectra (Figure 6c), the reduction reaction does not occur until lithiation of ≈175 mAh g^−1^ (i.e., ≈0.69 V). As shown in Figure 7c, the Cr^3+^ → Cr^2+^ (feature at 5995.8 eV) transition takes place before ≈675 mAh g^−1^ (≈0.44 V). Afterward, from ≈675 to 1200 mAh g^−1^, another conversion reaction from Cr^2+^ to metallic Cr (feature at 5989.0 eV) occurs until 0.01 V. Figure 6d shows that Fe^2+/3+^ is also reduced via a two‐step process, with the first reduction from lithiation at 200–525 mAh g^−1^ (0.66–0.51 V) and the second reduction from 525 to 825 mAh g^−1^ (0.51–0.36 V). According to Figure 7d, the first step is associated with the transition of Fe^2+/3+^ to Fe^2+^ (feature at 7119.7 eV), and the second step can be ascribed to the conversion of Fe^2+^ to metallic Fe. Similar to Cu^2+^, Ni^2+^ shows a single‐step conversion during lithiation, which occurs from 200 to 900 mAh g^−1^ (i.e., 0.66–0.31 V), as shown in Figure 6e. Figure 7e shows that, in this capacity range, the Ni^2+^ feature diminishes while the metallic Ni peak (at ≈8333.0 eV) continuously increases.

**Figure 8:**
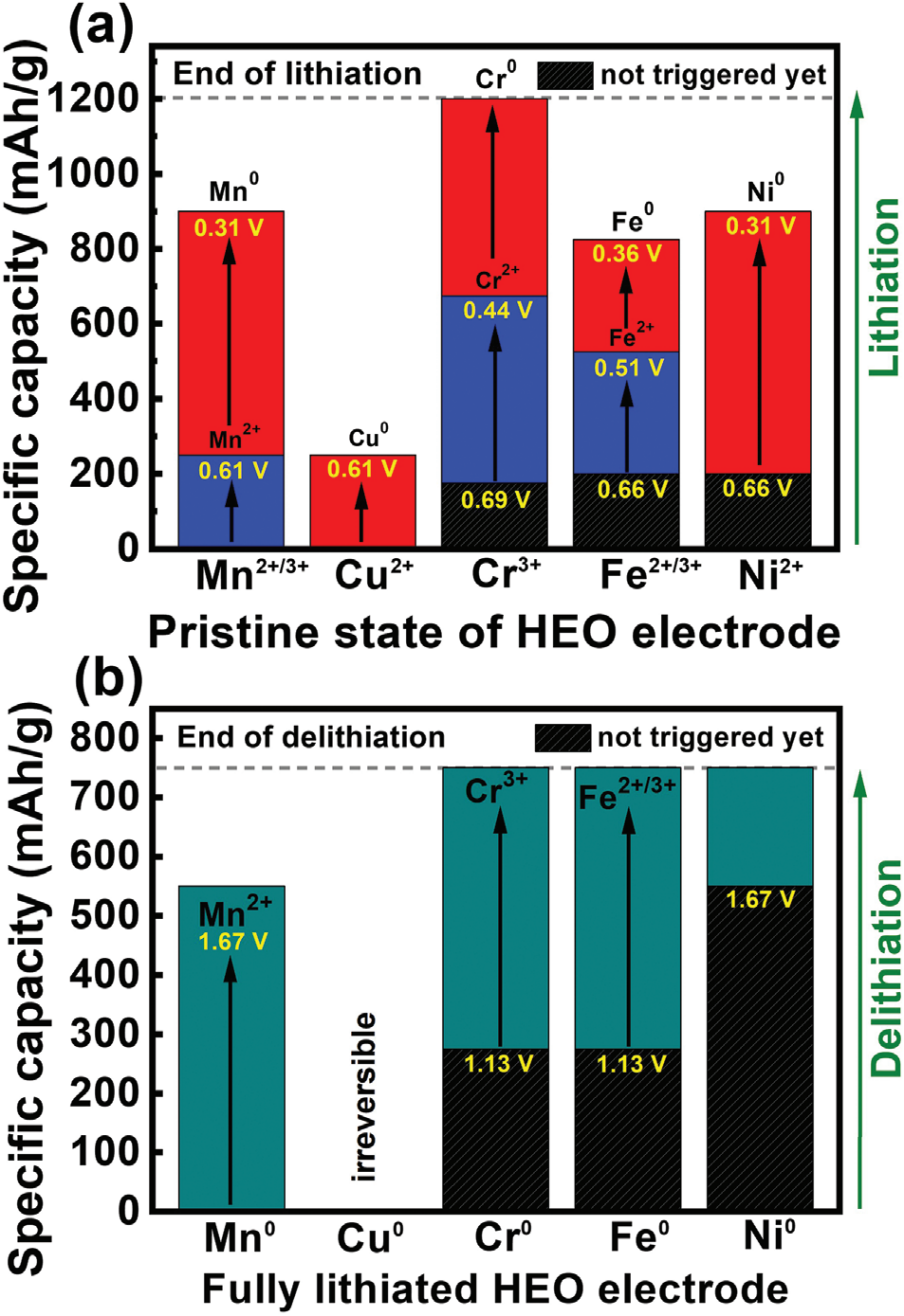
Summaries of transition behavior of all constituent elements in HEO electrode during the first a) charging and b) discharging.

**Figure** **8**a summarizes the transition behavior of the constituent elements in the (CrMnFeNiCu)_3_O_4_ HEO electrode during the first lithiation. Upon charging, the initial charge capacity is mainly attributed to the Mn^2+/3+^ → Mn^2+^ and Cu^2+^ → Cu^0^ reduction reactions. Then, the (Mn^2+^ and Mn^2+/3+^) → Mn^0^, Cr^3+^ → Cr^2+^, Fe^2+/3+^ → Fe^2+^, Fe^2+^ → Fe^0^, and Ni^2+^ → Ni transitions occur, leading to a charging potential plateau at ≈0.5 V. In the final stage of charging (after 900 mAh g^−1^), the Cr^2+^ → Cr° conversion plays a major role in charge‐compensating the Li^+^ storage.

Figure 6f–j shows the XAS K‐edge spectra of various elements during discharging from 0.01 to 3 V. Upon delithiation, the Mn spectra promptly begin to shift positively, indicating that the reoxidation can be driven by low overpotential. The monotonous energy shift proceeds until 550 mAh g^−1^ (≈1.67 V). As shown in the corresponding first‐derivative curves in Figure 7a, the metallic Mn peak decreases and the Mn^2+^ and Mn^2+/3+^ signals increase during delithiation. As exhibited in Figures 6g and 7b, there is almost no energy shift of the Cu spectra during delithiation, indicating that the Cu reaction is irreversible. The metallic Cu phase remains even at 3 V. For Cr (Figure 6h), a relatively large overpotential is needed to trigger the reoxidation reaction. After a delithiation capacity of ≈275 mAh g^−1^ (≈1.13 V), the Cr oxide forms, as shown in Figure 7c. After delithiation at 3 V, besides metallic Cr, Cr^3+^ is the dominant species according to the feature peak position of ≈5998.3 eV. As shown in Figure 6i, the reoxidation of Fe starts after a delithiation capacity of ≈275 mAh g^−1^. Figure 7d shows that the Fe oxide bump gradually increases in intensity, whereas the metallic Fe peak decreases until 3 V. At full delithiation, besides metallic Fe, Fe^2+^, and Fe^2+/3+^ states are observed. The energy shift of the Ni spectra during delithiation is shown in Figure 6j. Slight oxidation only occurs after a delithiation capacity of ≈550 mAh g^−1^ (≈1.67 V). As shown in Figure 7d, the metallic peak starts to decrease and the oxide bump marginally rises at 550−750 mAh g^−1^. The low reversibility of the Ni element in the HEO electrode is thus confirmed.

We also try to fit the XAS spectra using linear combination of standard metal and oxide spectra. However, because the nature of the HEO (i.e., the tetrahedral and octahedral metal sites are randomly occupied by Mn, Cu, Cr, Fe, and Ni), the chemical environments of individual elements are quite different from those of the standard samples. As shown in Figure S6 (Supporting Information), some spectra cannot be well fitted. However, the overall trend is still consistent with the results found in Figures 6 and 7. According to the data in Figure S6 (Supporting Information), certain amounts of metallic phase were found for all the elements after delithiation.

Figure 8b summarizes the above reoxidation processes during delithiation. The valence changes of Mn, Cu, Cr, Fe, and Ni during discharge are shown. In the initial discharge, the oxidation of metallic Mn is the main contributor to the measured capacity. After a delithiation capacity of ≈275 mAh g^−1^ (≈1.13 V), both Cr and Fe begin to participate in the oxidation reactions. The Cu reaction is the least reversible, probably due to its highest thermodynamic redox potential compared to those of the other elements, and thus provides little capacity. The overlapping oxidation processes of Mn, Cr, and Fe around 1.5 V explain the emergence of the discharge plateau and the distinct anodic CV peak (Figure 2a) in this potential region. A large overpotential (until ≈1.67 V) is needed to trigger Ni oxidation, which only contributes to capacity at the late stage of delithiation after ≈550 mAh g^−1^.

## 3 Conclusions

The synthesized cobalt‐free spinel (CrMnFeNiCu)_3_O_4_ LIB anode showed reversible capacities of 750 and 340 mAh g^−1^, respectively, at 50 and 2000 mA g^−1^. After 150 charge–discharge cycles, almost no capacity decay was found. The charge storage mechanism of this electrode was examined using operando quick‐scanning XAS with a high time resolution. In the pristine HEO, the cations are present as Mn^2+/3+^, Cu^2+^, Cr^3+^, Fe^2+/3+^, and Ni^2+^ and distributed at both the tetrahedral and octahedral sites. During the first lithiation, the Mn^2+/3+^ → Mn^2+^ and Cu^2+^ → Cu^0^ reactions contribute to the initial charge capacity in the potential sloping region. Then, the (Mn^2+^ and Mn^2+/3+^) → Mn^0^, Cr^3+^ → Cr^2+^, Fe^2+/3+^ → Fe^2+^, Fe^2+^ → Fe^0^, and Ni^2+^ → Ni^0^ transitions lead to a charging potential plateau at ≈0.5 V. Finally, the Cr^2+^ → Cr° conversion is responsible for the lithiation capacity from ≈900 to 1200 mAh g^−1^. Upon delithiation, the Cu reaction is hardly reversible and thus provides little capacity. Ni can be slightly reoxidized, but a large overpotential up to ≈1.67 V is required. The oxidation of Mn occurs first during discharge. The metallic Mn signal decreases and the Mn^2+^ and Mn^2+/3+^ signals increase upon delithiation. The Cr and Fe oxidation reactions start at a discharge capacity of ≈275 mAh g^−1^ (above ≈1.13 V). At full delithiation, besides metallic Cr and Fe, Cr^3+^, Fe^2+^, and Fe^2+/3+^ species are found. With an understanding of the detailed redox mechanism, more rational material design of HEO electrodes can be implemented for improving battery performance.

## 4 Experimental Section

### Synthesis of Spinel HEO Powder

The HEO sample was synthesized using a surfactant‐assisted hydrothermal method.^[^ ^18^ ^]^ Equimolar (1 mmol) Fe(NO_3_)_3_·9H_2_O (J. T. Baker, 99%), Ni(NO_3_)_2_·6H_2_O (Alfa Aesar, 98.5%), Mn(NO_3_)_2_·6H_2_O (Alfa Aesar, 98.5%), Cr(NO_3_)_3_·9H_2_O (Alfa Aesar, 98.5%), and Cu(NO_3_)_2_·2.5H_2_O (Alfa Aesar, 98%) were dissolved in 40 mL of deionized water, followed by the addition of 1.25 mmol (1‐hexadecyl)trimethylammonium bromide (Alfa Aesar, 98.5%) as a surfactant. 30 mmol urea (UniRegion Bio‐Tech, 99%) was then added with continuous stirring to form a homogenous solution. The solution was then transferred into a Teflon‐lined stainless steel autoclave for a hydrothermal reaction at 140 °C for 5 h. The precipitate was collected via centrifugation, repeatedly washed with deionized water and ethanol, and then dried at 60 °C in a vacuum oven for 12 h. The resulting material was annealed in air at 900 °C for 2 h to produce (CrMnFeNiCu)_3_O_4_ HEO powder.

### Preparation of Electrodes and Coin Cells

To prepare the electrode slurry, a mixture composed of HEO powder, Super P, and poly(vinylidene fluoride) at a mass ratio of 7:2:1 was dispersed in *N*‐methyl‐*2*‐pyrrolidone solution. The slurry was coated onto Cu foil (for charge–discharge tests) or graphite paper (for operando XAS study) using a doctor blade. The obtained electrodes were dried under vacuum at 85 °C for 10 h, roll‐pressed, and then cut to match the size of a CR2032 coin cell. An electrolyte that consisted of 1 m LiPF_6_ salt and ethylene carbonate/diethyl carbonate (1:1 by volume) solvent was used. Li metal foil and a glass fiber membrane were used as the counter electrode and separator, respectively. The assembly of the coin cells was performed in an Ar‐filled glove box (Vigor Tech. Co. Ltd.) with the moisture and oxygen content levels below 0.2 ppm.

### Material and Electrochemical Characterizations

The crystallinity of the sample was identified using XRD with a Cu K_*α*_ X‐ray source. The diffraction angle was scanned from 10° to 90° with a speed of 2° min^−1^. The HEO morphology was examined using SEM (JEOL 6701F). TEM (JEOL JEM‐2100F), EDS, and SAED were employed to study the material microstructure, chemical composition, and crystallinity, respectively. Inductively coupled plasma mass spectroscopy (ICP‐MS, Thermo‐Element XR) was utilized to quantify the constituent elements of the HEO.CV analysis was conducted with a potentiostat (Biologic VSP‐300) in a potential range of 0.01–3.0 V. The potential scan rate was 0.1 mV s^−1^. The charge–discharge properties of the HEO electrode were evaluated using an Arbin battery tester at 25 °C.

### Synchrotron X‐Ray Analyses

XAS and XRD were performed at beamlines TPS‐44A, TPS‐25A1, and TLS‐23A1 at the National Synchrotron Radiation Research Center in Taiwan. The XAS spectra were acquired in transmission mode. A quick‐scanning monochromator operated in on‐the‐fly mode was employed for the operando XAS study, in which a CR2032 coin cell with Kapton windows was charged and discharged at a specific current of 150 mA g^−1^. With a monochromator oscillation frequency of 2 Hz and the integration of 240 spectra, a 2 min time resolution (or a 5 mAh g^−1^ capacity resolution) of the operando XAS analysis was achieved. For the ex situ XAS and XRD measurements, the HEO electrodes were either i) lithiated at 0.01 V or ii) lithiated at 0.01 V and then delithiated at 3.0 V (vs Li^+^/Li). Then, the cells were disassembled within the glovebox, and the electrodes were removed for the analyses. A 15 keV incident beam (wavelength = 0.826 569 Å), a *q* range of 12–41 nm^−1^, and a sample‐to‐detector distance of 128.1 mm were used for the synchrotron XRD study.

### Statistical Analysis

The charge–discharge experiments of the HEO electrodes were repeated at least five times to ensure the validity. The reported capacities are the mean values, and the relative standard deviation is within 3%. For XAS data, the background subtraction, energy calibration, and spectrum normalization were conducted using the Athena software package. The Origin software was used for data analysis and processing.

## Conflict of Interest

The authors declare no conflict of interest.

## Supporting information

Supporting Information

## Data Availability

The data that support the findings of this study are available from the corresponding author upon reasonable request.
