# Supplementary material for: Charge–Discharge Mechanism of High‐Entropy Co‐Free Spinel Oxide Toward Li+ Storage Examined Using Operando Quick‐Scanning X‐Ray Absorption Spectroscopy
Source: Adv Sci (Weinh). 2022 May 26;9(21):2201219. doi: 10.1002/advs.202201219 (PMC9313486; doi:10.1002/advs.202201219)
Supplement: Supplementary file 1 — Supporting Information [file ADVS-9-2201219-s001.pdf]

ADVANCED SCIENCE
Open Access

Supporting Information

for *Adv. Sci.*, DOI 10.1002/advs.202201219

Charge–Discharge Mechanism of High-Entropy Co-Free Spinel Oxide Toward Li^+ Storage Examined Using Operando Quick-Scanning X-Ray Absorption Spectroscopy

Xu-Feng Luo, Jagabandhu Patra, Wei-Tsung Chuang, Thi Xuyen Nguyen, Jyh-Ming Ting, Ju Li, Chih-Wen Pao and Jeng-Kuei Chang**

Supporting Information

Charge-Discharge Mechanism of High-Entropy Co-Free Spinel Oxide Toward Li^+ Storage Examined Using Operando Quick-Scanning X-ray Absorption Spectroscopy

Xu-Feng Luo, Jagabandhu Patra, Wei-Tsung Chuang, Thi Xuyen Nguyen, Jyh-Ming Ting, Ju Li, Chih-Wen Pao, Jeng-Kuei Chang**

Table S1. Chemical composition of $(CrMnFeNiCu)_3O_4$ HEO powder examined using ICP-MS.

Elements	Weight %
Cr	22.75
Mn	20.95
Fe	23.27
Ni	21.93
Cu	11.10

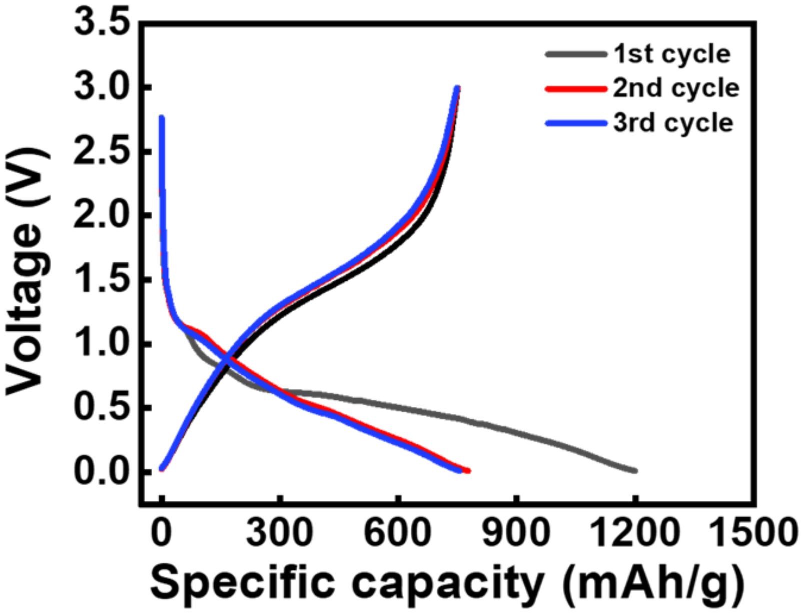

Figure S1. Initial three charge-discharge curves of HEO electrode recorded at 50 mA g^{-1}.

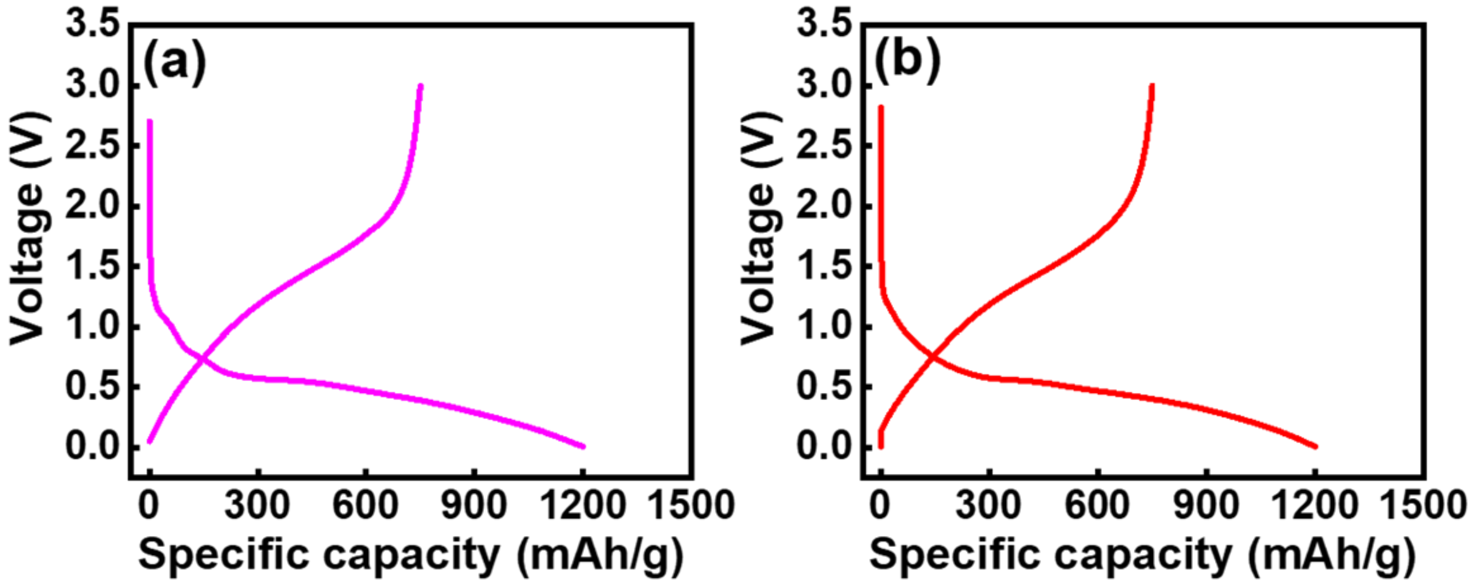

Figure S2. Charge-discharge curves of HEO electrodes with (a) Cu foil and (b) graphite paper current collectors measured at 150 mA g^{-1}.

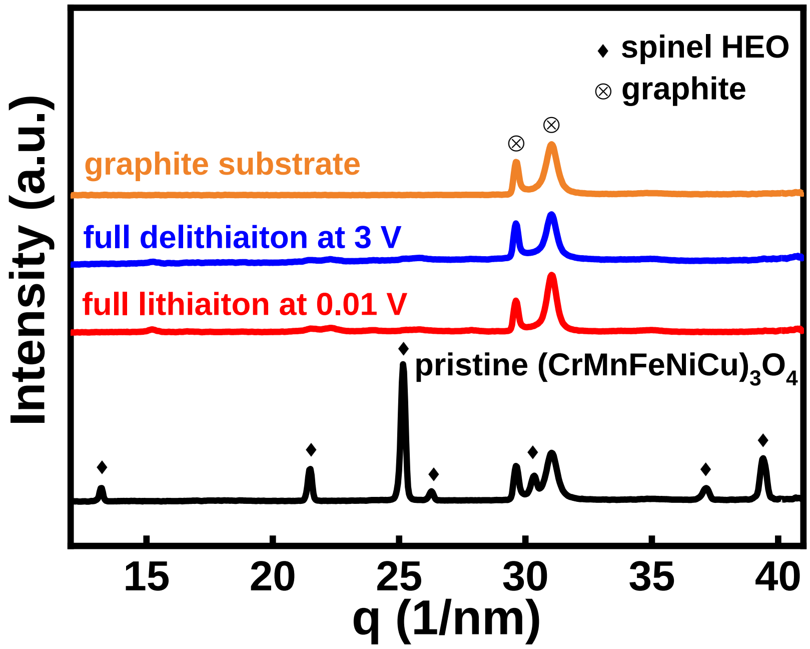

Figure S3. XRD patterns of $(CrMnFeNiCu)_3O_4$ electrodes after lithiation and delithiation. The pristine electrode and graphite substrate (current collector) data are also shown for comparison.

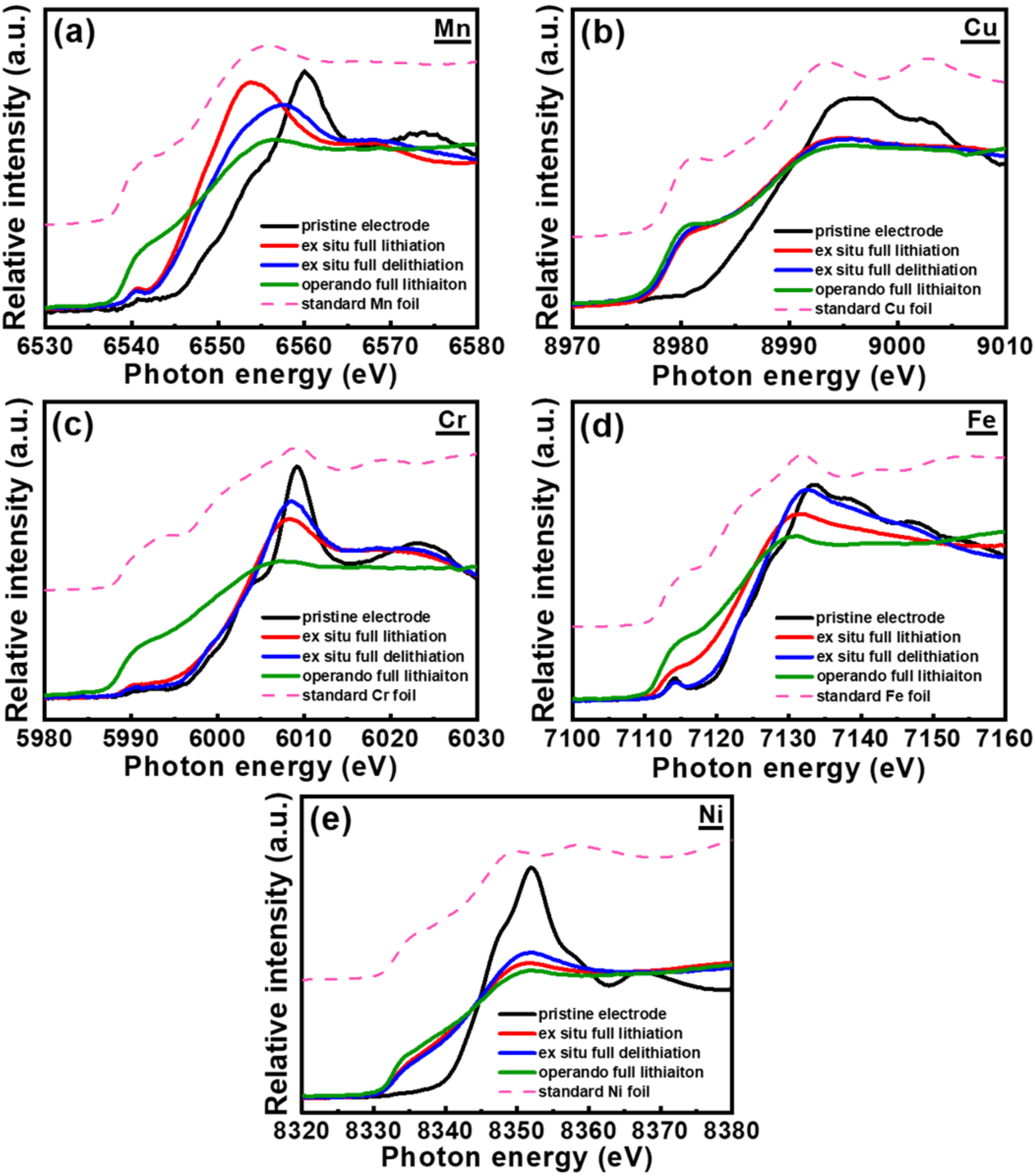

Figure S4. Ex situ (a) Mn, (b) Cu, (c) Cr, (d) Fe, and (e) Ni XAS spectra of HEO electrodes at pristine, lithiation, and delithiation states. Operando spectra measured at 0.01-V lithiation are shown for comparison.

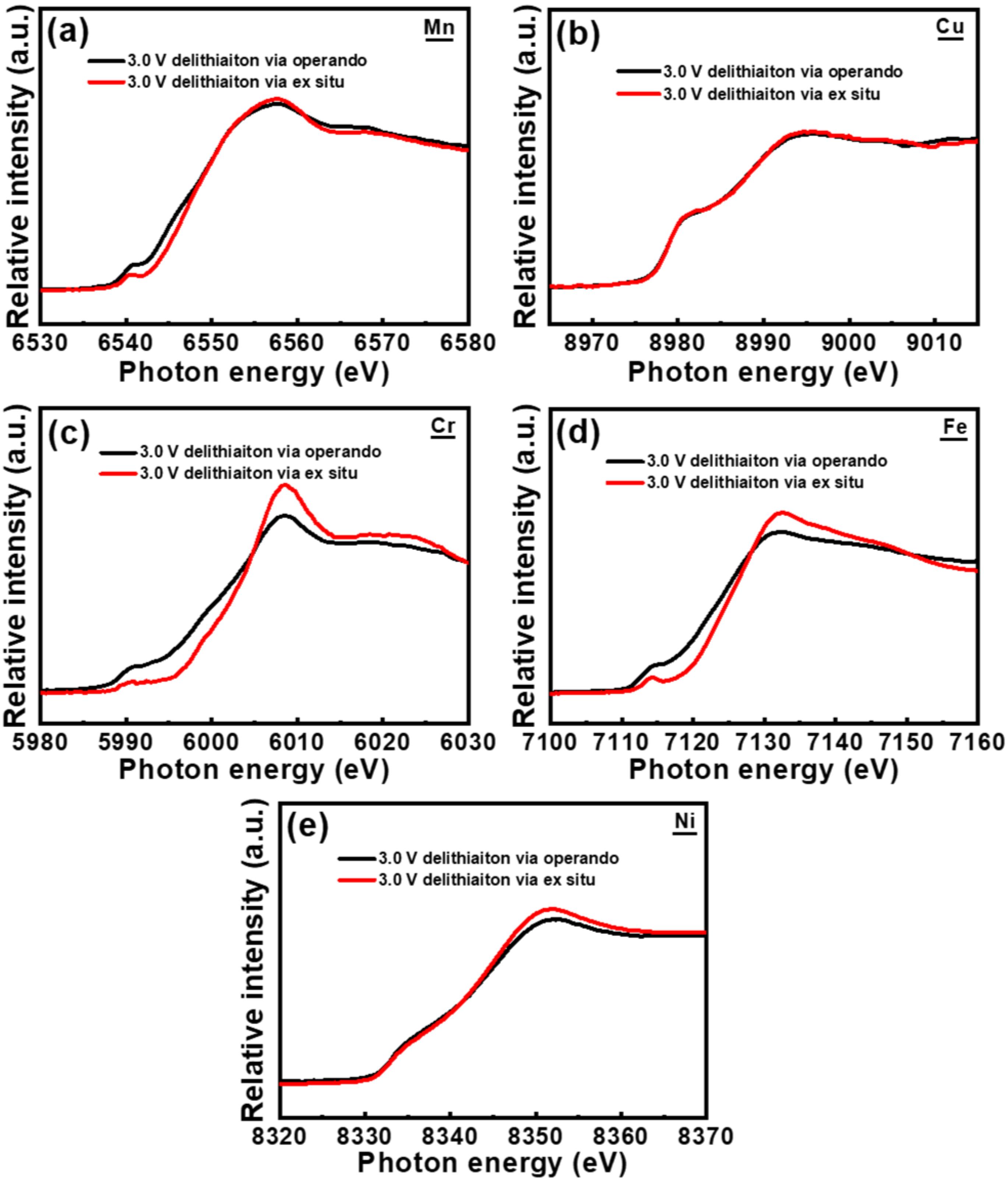

Figure S5. Comparison between (a) Mn, (b) Cu, (c) Cr, (d) Fe, and (e) Ni K-edge XAS spectra of delithiated HEO electrodes measured using ex situ and operando methods.

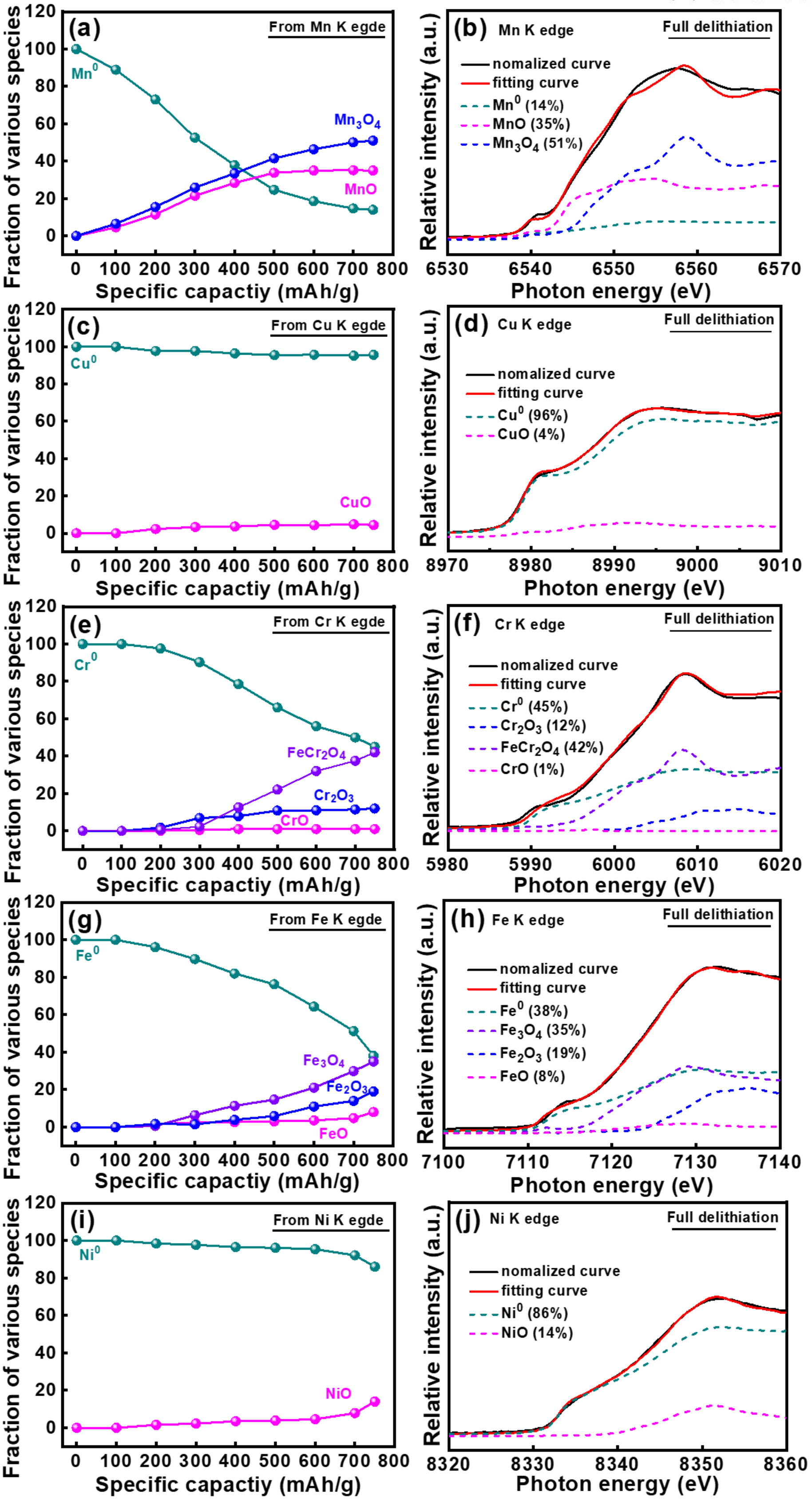

Figure S6. Linear combination fitting results of (a, b) Mn, (c, d) Cu, (e, f) Cr, (g, h) Fe, and (i, j) Ni K-edge XAS spectra. Standard metal and oxide spectra are used for data fitting. Right figures show fitting data at full delithiation state.
